# ‘Gearing Up’ to improve interprofessional collaboration in primary care: a systematic review and conceptual framework

**DOI:** 10.1186/s12875-016-0492-1

**Published:** 2016-07-20

**Authors:** Gillian Mulvale, Mark Embrett, Shaghayegh Donya Razavi

**Affiliations:** DeGroote School of Business, McMaster University, 4350 South Service Road, Rm 421, Burlington, ON Canada L7L 5R8; Faculty of Health Sciences, McMaster University, CRL Bulding 282, 1280 Main Street West, Hamilton, ON Canada L8S 4K1

**Keywords:** Interprofessional collaboration, Primary care team, Policymaking, Conceptual model

## Abstract

**Background:**

Interprofessional Primary Care Teams (IPCTs) have been shown to benefit health systems and patients, particularly those patients with complex care needs. The literature suggests a wide range of factors that may influence collaboration in IPCTs, however the evidence base is unclear for many of these factors. To target improvement efforts, we identify studies that demonstrate an association between suggested factors and collaborative processes in IPCTs.

**Methods:**

A systematic review of 25 years of peer-review literature was conducted to identify studies that test associations between policy, organizational, care team and individual factors, and collaboration in IPCTs. We searched Medline, ProQuest subject, ProQuest abstract, CINAHL, HealthSTAR, and Embase electronic databases between January 1990 to June 2015 and hand-searched reference lists of identified articles.

**Results:**

The electronic searches identified 1421 articles, nine of which met inclusion criteria.

Eighteen factors were significantly associated with collaboration in at least one article.

We present the findings within a proposed conceptual model of interrelated ‘gears’. The model offers a taxonomy of factors that policy makers (macro gear), organizational managers (meso gear), care teams (micro gear) and health professionals (individual gear) can adjust to improve interprofessional collaboration in IPC teams. Thirteen of the eighteen identified factors were within the micro gear, or team level of decision-making. These pertained to formal processes such as quality audits and group problem-solving; social processes such as open communication and supportive colleagues; team attitudes such as feeling part of the team; and team structure such as team size and having a collaboration champion or facilitator. Fewer policy (eg governance), organizational (eg information systems, organizational culture) or individual (eg belief in interprofessional collaboration care and personal flexibility) level factors were identified.

**Conclusions:**

The findings suggest that individual IPCTs have opportunities to improve collaboration regardless of the organizational or policy context within which they operate. Evidence supports the importance of having a team vision and shared goals, formal quality processes, information systems, and professionals feeling part of the team. Few studies assessed associations between collaboration and macro and meso factors, or between factors across levels, which are priorities for future research.

**Electronic supplementary material:**

The online version of this article (doi:10.1186/s12875-016-0492-1) contains supplementary material, which is available to authorized users.

## Background

The World Health Organization indicates that collaborative practice by multiple health care workers strengthens health systems, patient satisfaction, acceptance of care, and improves patient outcomes [1]. Policy-makers in many countries have focused attention on advancing care delivery and enhancing collaboration within interprofessional primary care teams (IPCTs) [2–10], with a particular view to supporting the needs of populations with different chronic diseases [11–13]. For example, in the United Kingdom, multidisciplinary care teams are integral to the personalized care plan developed by the Department of Health to better address the needs of the ageing population and those living with long-term conditions [14]. Similarly, in the United States, IPCTs are a key component of the patient-centred medical home [15], a care delivery model favoured for improving quality and decreasing cost of care [16]. In Canada, diverse approaches have been implemented, including reforming policy factors such as funding, professional regulation and remuneration to encourage primary care physicians to restructure individual practices toward interprofessional care [17], and the creation of networks of professionals from different disciplines who collectively meet the needs of different patients [3, 4, 18]. In addition, a national interprofessional competency framework has been developed which points to the importance of six key domains: interprofessional communication; patient/client/family/community-centred care; role clarification; team functioning; collaborative leadership; and interprofessional conflict resolution [19]. Yet, recent work suggests that teams vary in the extent and effectiveness of collaboration [9, 20]. If IPCTs are to realize their full potential, policy makers, health care managers, team leads and members need evidence on how to best improve collaboration in interprofessional teams [21].

The existing academic and policy literature suggests a broad range of factors both internal and external to the health care team that influence how well IPCTs collaborate [4, 22–27]. In the conceptual literature on interprofessional practice, collaborative factors are often portrayed using an input–output-processes approach [24, 27] that originated in the human resources literature [28]. This approach defines inputs as contextual variables within the team, the organization or the broader policy environment. The process component of the approach includes intragroup processes and behaviours that occur in teams that influence the team’s overall collaborative performance (the output). Collaborative performance in turn influences outcomes, such as patient health status and satisfaction, quality of care, cost-effectiveness, professional wellbeing, and willingness of team members to work together in the future [24].

Alternative models of IPC collaboration have emphasized similar factors. In Sicotte and colleagues’ (2002) model of IPCTs, contextual variables (eg characteristics of program managers and structural characteristics of the program) and intragroup process variables (eg belief in benefits of collaboration, work group design characteristics, social integration within groups) determine the intensity of collaboration, with the nature of the task that relates to patient need as a mediating variable [27]. Lemieux-Charles (2006) expands the inputs to include social and policy context, a wider array of organizational context variables, additional detail on the patient and disease type, as well as clarity of rules and procedures [24].

Using many of the factors identified in the input–output-processes approaches, Mulvale and colleagues organized contextual factors as an interrelated set of policy (macro), organizational (meso), team (micro) and individual factors. This perspective helps recognize where opportunities exist for decision-makers at each level to encourage more effective collaboration in IPCTs [25]. Macro factors include professional regulation, professional education, funding, and provider payment schemes. Meso factors include organizational structure, rewards, and information systems [4, 24, 27, 29–32]. Micro factors include processes based on mutual trust, and power-sharing that reflects knowledge and experience rather than titles [33–35]. Individual factors include maturity in one’s profession, and attitudes toward collaborative practices [36]. There are interactions among factors at all three levels, for example, professional regulation influences information systems at the organizational level, understanding of professional roles at the micro level and confidence in one’s profession at the individual level.

Although these models capture many factors that have been suggested as having a relationship to collaboration, the statistical evidence for these associations is unclear. A systematic review by Zwarenstein and colleagues (2009) found few intervention studies to improve collaboration in IPCTs and concluded that the association of many of these factors with collaborative processes should be viewed as “promising” rather than “proven”([37] p.8). This suggests a need to revisit the quantitative evidence base to identify the findings of more recent intervention or observational studies that can support efforts to improve collaboration in IPCTs [9, 15, 37, 38]. Reeves et al. (2010) further suggest that the theoretical base is underdeveloped and call for more empirically-based theory [39], while Hall, Weaver, and Grasseau (2013) suggest the need to weave together elements of many elements of existing theories [40].

The primary objective of this study was to systematically review the literature to identify factors that have been shown to have a statistically significant association with collaboration in IPCTs. A secondary objective was to position the identified factors within a conceptual model that integrates earlier models and reflects new thinking about dynamic approaches to improving collaboration in IPCTs. The overarching goal was to create a taxonomy of factors that could assist decision-makers at various levels (policy, organization, health care team, individual provider) in identifying promising areas in which to take action to improve IPCT collaboration and whether or not there was statistical evidence for each of the suggested factors.

### Proposed “Gears” conceptual model

**Fig. 1:**
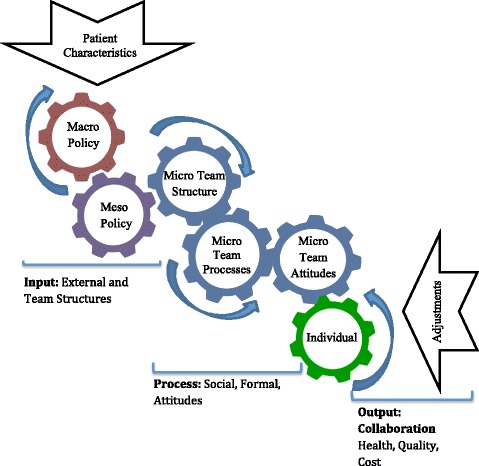
Gears Model of Factors Affecting Interprofessional Collaboration within IPCTs. This proposed dynamic “gears model” draws together a variety of factors that have been suggested to affect interprofessional collaboration within IPCTs by integrating the input-processes-output approach, the macro-meso-micro-individual model, and the insights gained from literature on the dynamic nature of health care teams

In Fig. 1, we present a conceptual model that will be used in interpreting the findings of the systematic review. This proposed dynamic “gears model” integrates the input-processes-output approach, the macro-meso-micro-individual model, and the insights gained from recent literature on the dynamic nature of health care teams to point to the ‘fluid’ nature of the current health care environment [41], which “constantly shifts, changes and requires rapid modifications of relationship strategies between participants” ([40], p.75). The model suggests that the relationship among internal and external factors is in a state of flux in response to changing policy and organizational contexts and individual patient needs [42]. For example, in many jurisdictions, policies to promote new models of care are introduced, but health professionals may face uncertainty with respect to how long the program will be supported, and these uncertainties can create concerns about the future roles of different team members that may affect collaboration [35]. In addition, patients with multiple chronic conditions [41] such as mental health problems [12] or rehabilitation patients [13] may require collaboration from many more health professionals than patients with less complex needs. This suggests changing team composition for different patients.

Individual patient needs and preferences determine whether a response by an individual provider or by an interprofessional team is appropriate. If a team approach is required, collaborative performance will be influenced by macro, meso, micro and individual contextual factors (inputs) and process factors that include social and formal processes, team attitudes and the traits and attitudes of team members (individual factors). Some contextual factors are external to the team, such as policy and organizational factors, while team structure is internal. These various factors are represented as a set of gears that work together to determine the collaborative performance of the team as the output, which in turn contributes to outcomes in terms of patient health outcomes, quality of care, professional experience, cost and efficiency. Monitoring of performance measures can trigger adjustments in the gears that influence collaborative processes. Macro factors (eg regulations regarding scopes of practice, funding mechanisms, provider payment) change less frequently but have widespread impact when they do change. Meso level factors such as organizational culture, team structure, leadership, training and rewards may change more often and affect multiple teams in an organization. The micro level gears affect the individual team, but we would expect many adjustments as continuous improvements are made in patient-centered teams. The gears themselves would be adjusted to reflect fine-tuning of collaborative processes based on adaptive day-to-day learning, and responses to issues raised at team meetings, quality audits, annual performance feedback and evaluation results as indicated by the ‘Adjustment’ arrow which flows from collaboration as an output of the model as well as patient, quality and cost outcomes.

## Methods

The Preferred Reporting Items for Systematic Reviews and Meta-Analyses (PRISMA) was used as a reporting guide [43] for the systematic review of studies that measure associations among various factors and collaboration in IPCTs. A protocol including research question, objectives, definition of interprofessional collaboration, identification of key words and information sources, and eligibility criteria were specified and documented in advance.

### Information sources

We searched six electronic databases (Medline, ProQuest subject, ProQuest abstract, CINAHL, HealthSTAR, and Embase) for peer reviewed scholarly publications (Fig. 1). The 25-year review covered the period of January 1990 to June 2015.

### Eligibility criteria

We adopt Zwarenstein, Goldman, and Reeves’ (2009) definition of interprofessional collaboration as “the process in which different professional groups work together to positively impact health care,” ([37] p.2) while recognizing that “interprofessional collaboration also involves issues that arise due to different professionals working together, such as problematic power dynamics, poor communication patterns, lack of understanding of one’s own and others’ roles and responsibilities, and conflicts due to varied approaches to patient care” ([37] p.2). The literature typically describes a team as consisting of two or more individuals; however, for the purposes of our review we define IPCTs as teams that include members of at least three different health professions that work together to meet the multiple primary care needs of a target patient population. This enabled our search to go beyond the extensive literature about the traditional nurse-physician dyad or physician-physician assistant dyad for example, to consider teams whose members had training from three or more professional backgrounds. In order to be as comprehensive as possible in our review of the literature, we include papers that focus on collaboration in primary care teams (ie delivering care to individuals) and in primary *health* care teams (ie delivering care to individuals and offering population health interventions) [44]. We focus on studies that measure the relationship between particular factors and the effectiveness of interprofessional collaboration as perceived by members of the IPCT. This focus recognizes that interprofessional collaboration is an intermediary result that may or may not be linked to end results such as the improved health of the patient, greater efficiency or reduced costs, or improved quality of care [27]. We did not require the included studies to have a particular outcome focus, only that they examine associations between various factors and interprofessional collaboration, as either an intermediate or end outcome. We consider factors typically associated with the policy (macro), organizational (meso), team (micro) and individual contexts.

Eligibility criteria for the electronic search included studies of any design type that were: (1) published in peer review journals; (2) focused on IPCTs; (3) operated in a community primary care setting; and (4) reported on the significance of associations between particular factors and the effectiveness of interprofessional collaborative processes. We did not assess the magnitude of these associations because we were unable to make meaningful comparisons across studies due to marked differences in study designs. Our objective was to identify factors that were found to be significantly associated with IPCT collaboration in the identified studies. We adopted a number of broad search terms to capture notions of ‘collaboration.’ We focused on studies that refer to the ‘quality’ or ‘intensity’ of collaborative processes as defined in each article. For example, Sicotte and colleagues (2002) [27] refer to intensity of interdisciplinary collaboration, while others [24, 45] refer to team effectiveness, still others to team climate [46] or teamwork [47, 48].

### Search terms

We used a combination of key words related to “interprofessional” (eg “interdisciplinary”, “multidisciplinary”, “interprofessional”) and “primary care” or “primary health care” alone and in conjunction with specific terms related to collaboration, team functioning and effectiveness (“team dynamic”, “team function”, “collaboration”, “participation”, “innovation”, “decision making”, “communication”, “technology”, “information technology”, “group process”, “cooperation”, “team culture”, “power”, “trust”, “practice guideline”, “communication protocol”, “accountability”, “conflict management”, “mutual respect”, “workload”, “training”, “professional support”), and “regulatory factors” (eg “scope of practice”, “professional regulation”). A sample search string is found in Additional file 1.

### Abstract review and data extraction

The abstract review was performed by two of the authors (ME & SDR). The full research team reviewed any abstracts deemed unclear for inclusion. A data extraction sheet was developed that captured: authors and date of publication, descriptions of the study (study aim, method, setting/model, team composition, and sample size), findings in terms of statistical tests of associations between particular factors and effectiveness of IPCT collaboration. All members of the team contributed to data extraction and verification using the data extraction template.

### Quality assessment

Two authors (ME & SDR) independently performed a quality assessment [49] of all articles in order to determine the methodological quality, and the full team met to discuss discrepancies until consensus was achieved.

## Results

**Fig. 2:**
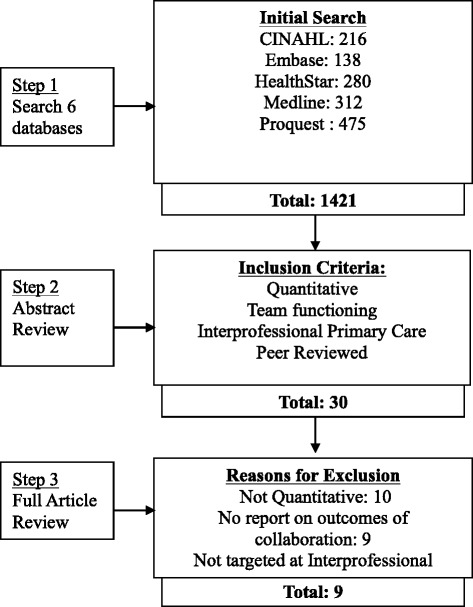
Search Strategy and Results. This figure documents the number of identified articles at each stage of the search

The search of electronic databases yielded 1421 articles, with 30 remaining following abstract review (see Fig. 2), and 9 articles remaining following full text review. Reference checking did not reveal additional articles that met inclusion criteria. Most of the articles that were excluded were strictly qualitative in nature, and/or did not investigate interprofessional collaboration.

Table 1 provides an overview of the identified studies in terms of study aims and methods, main findings and quality assessment scores. The articles covered primary care teams in several jurisdictions: Canada (Quebec, Alberta), Spain, the United Kingdom (Scotland, England), the United States and Puerto Rico. Six of the included articles described studies that used survey methods to explore factors related to perceived collaboration in IPCTs [27, 45, 46, 50–52]. The remaining three articles were intervention studies [48, 53, 54]. Team collaboration was the primary or one of several primary outcomes specified in three studies [46, 48, 53], in other studies it was an intermediate outcome [27, 45, 50–52, 54]. Our analysis focused only on estimates of associations between the specified factor(s) and collaboration in IPCTs.

**Table 1:** Results of Systematic Review

(1) Authors	(2) Study Aim And Principal Outcome(s)	(3) Outcome(s)	(4) Method	(5) Setting/Model	(6) Team Composition	(7) Sample	(8) Study Findings: Factor and its Relationship to IPC Collaboration Processes*	(9) Significant Factors	(10) Quality Score
1. Sicotte et al. (2002)	To understand Interdisciplinary collaboration among groups of professionals.	Interdisciplinary co-ordination Interdisciplinary care sharing activities	Survey, Questionnaire of health professionals within teams. Regression analysis to model factors associated with two separate outcome measures: (i) interdisciplinary coordination and (ii) interdisciplinary care sharing activities.	Community Health Care Centers (CLSCs) in Quebec, Canada	Varies. May include physicians, nurses, social workers, physical therapists, occupational therapists and psychologists	*N* = 157	Formal assessment of care quality (S) Beliefs in the benefits associated with interdisciplinary collaboration (S) Social integration within work groups” (S) “Level of conflicts associated with interdisciplinary collaboration” (S) Unique clinical data form (S) *R* ^2^ = .59 for interdisciplinary collaboration *R* ^2^ = .72 for interdisciplinary care-sharing activities program manager (NS) structure (NS)	Quality Audit/process Belief in IPC teamwork Feeling Part of the Team Levels of Conflict Information Systems	12
2. Gene-Badia et al. (2007)	Assess components of primary health care output using Confirmatory Factor Analysis	Scientific-Technical quality Team coordination	Survey of IPC teams. A confirmatory factor analysis was carried out to determine factors associated with team coordination.	Primary Care Teams in Catalunya, Spain.	Varies. Includes physicians, nurses, clerical staff.	*N* = 213	Support from supervisors (β =0.676) (NS) Support from colleagues (β = 0.859) (S) Work feedback (β =0.616) (NS) Proposal listened to and applied (β = 0.325) (NS)	Supportive Colleagues	14
3. McLean et al. (2005)	Impact of Quality Practice Award (QPA) on teamwork in IPC teams.	Teamwork in the practice	Survey of IPC team members who had completed QPA process to determine to what extent their perception of teamwork had increased by completing the QPA.	Primary Health Care Teams, Scotland.	Varies. GPs, practice nurses, community nursing staff, administrative staff.	*N* = 158	Completing the quality accreditation process led to perceived improvement in IPC collaboration across all professional groups. (*p* = 0.000).	Quality audit/process	12
4. Poulton, & West (1999)	Examine the relationship between team structure and processes and team effectiveness.	Team Work Organizational efficiency Health care practice Patient-centred care	Survey of health professionals in IPC teams. Correlations and regression analysis between team processes and structure measures and team effectiveness measures (emphasis here on team work measure).	Primary care practices, UK.	Varies. GPs, health visitors, district nurses, practice nurses, receptionists, midwives, counselors, community psychiatric nurses.	*N* = 528	Shared objectives (*r* = 0.51, *p* < 0.001). Support for innovation (*r* = 0.41, *p* < 0.01) Quality emphasis (*p* < 0.05) Participation (NS) Team Size (NS) Team Tenure (NS) Fundholding Status (NS)	Team Vision/Goals Support for innovation Quality audit/process	12
5. Hern, et al; (2009)	Assess whether the introduction of patient care management teams improves continuity of care, office efficiency and communication.	Continuity of care Office Efficiency Team Communication	Survey of IPC team members to evaluate perceived effectiveness of changes over time (9 and 20 months) following introduction of patient care management team.	IPC residency clinic, Chicago, Illinois, U.S.A.	Faculty, residents, RNs, Medical Assistants, Clerical Assistants, Medical Records Staff	*N* = 62	Intervention: changes to team structure and size formalization of monthly team meetings, introduction of electronic message management system and common electronic system for lab results Scores on team communication increased after 9 months and were maintained at 20 months. (*p* < .05). (S)	Team size Team Meetings Information Systems	12
6. Bower et al. (2003)	Assess whether practice structure influences team processes and whether both structure and process predict team outcomes.	Self-Rated Team Effectiveness Innovation Various Chronic Disease Management Measures	Self-report measures and questionnaires among staff within and attached to 42 general practices. Regression analyses. Focus here is on impact on team climate measure.	General practices in England.	Doctors, nurses, non-medical clinical staff, administrative staff	*N* = 802	Singlehanded practices (vs. partnerships) were associated with better team climate (as measure of team process). (β = 2.38) (S)	Governance	13
7. Shortell (2004)	Assess if interprofessional collaboration is related to quality improvement, and to assess differences in perceived team effectiveness.	Perceived Team Effectiveness	Surveyed team members as part of the U.S. National evaluation of the Improving Chronic Illness Care program. Regression analysis of relationship between a number of factors on perceived team effectiveness (includes IPC collaboration).	Chronic care teams (from 21 US states and Puerto Rico	Varies. Not specified.	*N* = 40 teams	organizational team culture balance (β = 3.10, *p* < 0.10), patient satisfaction as focus (β = 0.49, *p* < 0.05), presence of a team champion (β = 0.69, *p* < 0.01) Team size (β = -0.06,) *p* < 0.10) *R* ^2^_adj_ = 0.40	Organizational Culture Team Vision/Goals Champion/Facilitator Team Size	13
8. Goni (1999)	Assess the relationship between team design, individual characteristics and team performance.	Team Performance Team Reliability	Surveys of IPC members and administrative data. Two groups of teams were identified based on a cluster analysis: reliable and worse teams. Differences between groups attributable to each factor was estimated using one-way ANOVA.	Primary health care teams (PHCTs) Navarre, Spain	Doctor, pediatrician, nurse, social work, administrative staff.	*N* = 256	common goals (*p* < 0.01) empowerment (feeling part of team, team has ability to overcome problems (*p* < 0.01) communication (*p* < 0.01) flexibility (*p* < 0.01) recognition (*p* < 0.01)	Team Vision/Goals Feeling Part of Team Group problem-solving Open communication Flexibility Recognition	9
9. Dieleman (2004)	Evaluate the impact of team care on providers’ attitudes.	Provider attitudes toward team activities (job satisfaction, role recognition, experience in team, quality of care)	Questionnaire used in pre and post-test design.	General primary care setting, Alberta, Canada.	Pharmacists, physicians, nurses.	*N* = 22	Better functioning teams were more satisfied with decision-making process and decisions (*p* = 0.03).	Decision-making processes	13

*‘S’ = Statistically significant and ‘NS’ = not statistically significant as reported by study authors

Column 8 of Table 1 lists the factor(s) reported on in each study identified in the review. Different studies tested associations for different factors with different model specifications. Based on our understanding of what was reported, sometimes similar constructs were given different labels by different authors. In column 9 of Table 1 we specify the factors identified in the review, using a common label where factor descriptors differed across articles. A total of 18 unique factors were identified across the nine studies. Having a team vision or shared goals and objective(s) [45, 51, 52] and the use of quality audits or processes [27, 45, 48] were identified in three articles each.^1^ The size of the team [52, 54], use of information systems [27, 54] and feeling part of the team [27, 51] were identified in two studies each. Many factors were identified a single study.

**Fig. 3:**
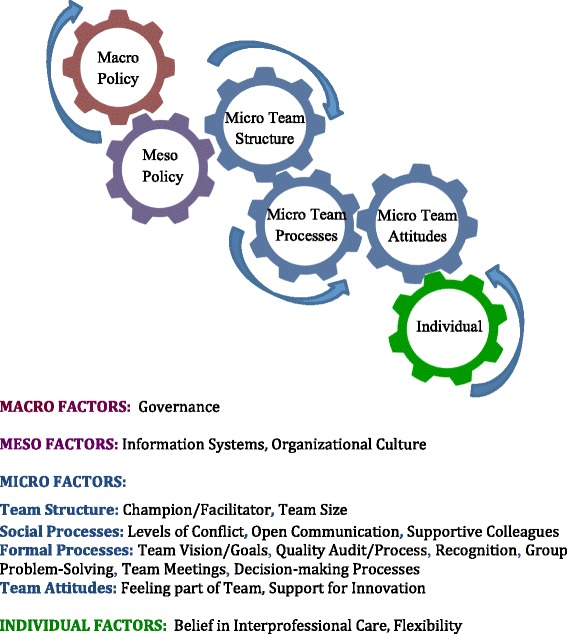
Factors Identified as Being Associated with Collaboration in IPCTs. This figure lists the factors identified as being significantly associated with interprofessional collaboration in IPCTs based on the systematic review of the published literature, shown within the gears model

In Fig. 3 we place the factors identified in the review within the gears model. We focus on the context and process factors that are associated with collaborative performance in Fig. 1. Thirteen of the eighteen identified factors were within the micro gear. We group these as formal processes, social processes, team attitudes and team structure. The six formal team processes were: setting a team vision or goals [45, 51, 52], having a focus on quality through audits or other processes [27, 45, 48], having formal recognition from supervisors 2 [51], processes for group-problem solving [51] and decision-making [53], as well as holding interprofessional team meetings [54]. Social processes included having low levels of conflict in the team [27], supportive colleagues [50], and open communication [51]. Attitudinal factors were feeling part of the team [27, 51] and feeling that there is support for innovation within the team^3^ [45]. In terms of team structure, having a team champion [52], and team size [52] were associated with collaboration. A negative but nonlinear association was found between collaboration and team size, suggesting that as teams grew beyond an optimal size, collaborative processes were less effective [52].

There were two factors within the meso gear that were identified in the review: having formal information systems [27, 54] and an organizational culture that had a balance with respect to group culture, hierarchy, and focus on efficiency and achievement [52]. Two factors were identified within the individual gear: believing in the concept of interprofessional collaboration [27], and flexibility [51]. Governance was the only macro factor among the identified articles, although this could also be considered a meso factor depending on the context at which decisions about governance are made (ie state or practice level). Bower and colleagues [46] found that having a single-handed governance structure was positively associated with team climate, but questioned whether governance was acting as a proxy for another variable that could be more easily targeted in interventions to improve IPCT collaboration.

## Discussion

Our review identified a number of studies that have measured associations between collaborative processes in IPCTs and structural and process factors; however, there is limited statistical evidence for many factors suggested elsewhere in the literature. This may reflect lack of research in these areas, rather than lack of significance. Where quantitative studies exist, the suggested factors were most often found to be significantly associated with collaborative processes in at least one study, although in a few cases there was conflicting evidence across studies. When the findings are viewed within the proposed ‘gears’ conceptual model, empirical support is found for some factors within each of the macro, meso, micro and individual gears [25, 26, 38]. The majority of factors identified in the review were within the micro gear, corresponding to team level decision making.

The broad categories of factors and their relative importance within the gears model as portrayed here are consistent with reviews that primarily identified qualitative studies that examine factors that influence IPCT collaboration [4, 20, 55, 56].

Based on the existing evidence, policy makers may want to examine the influence of alternative governance structures for healthcare practices in terms of how they might influence the extent to which team members feel they are part of the team. For example, lack of input to board decision-making and part-time status of some health professionals led to feelings of lack of power in a study of IPCT collaboration in Ontario, Canada [26]. Health care organizations may want to reflect on how their organizational culture and information systems influence collaborative processes. Health care teams may benefit from examining formal and social processes to set a common vision, ensure all professionals feel part of the team and are comfortable in putting forward their ideas to improve teamwork and care delivery. There is also some evidence that when it comes to team structure, the size of the team matters, and having a team champion or facilitator can focus efforts to improve collaboration in IPCTs. Furthermore, team members may want to examine their personal commitment to interprofessional care and their own flexibility when working with other professionals. These individual characteristics are also important to consider in recruitment decisions.

The proposed gears model helps to advance conceptual thinking by bringing together existing approaches within an interactive, dynamic system of factors that can be driven by different policy and health system actors. The model uses the metaphor of interrelated gears to indicate that multiple and reciprocal relationships may exist among the factors that are difficult to separate. The metaphor suggests that external and internal factors do not operate in isolation. For example, a larger team may have a wider range of clinical skills, but will only be able to take advantage of these if the team environment is conducive to collaboration through micro factors such as openness to innovation and open communication [46]. The model also suggests that it may be necessary to adjust factors at multiple levels to support improved collaborative processes. This suggests that improving collaboration needs to occur on an ongoing basis within a dynamic, changing system. The model further supports the need for partnership among players at the individual, team, organization and policy levels to create smoothly running care teams that are responsive to individual patient needs. In the case of an IPCT, for example, problems in information systems at the organization level may be overcome through more team meetings and fostering more open communication at the team level. This is consistent with the ‘workarounds’ that health professionals discussed as necessary to overcome structural barriers to collaboration in a study of scopes-of practice and innovative models of care delivery in Canada [57].

At the same time there are several limitations to this analysis that must be considered. While statistical significance is one consideration when assessing the empirical basis of suggested factors, the magnitude of association with collaboration is also important, but was not comparable across studies. In addition, only a limited number of studies were identified in the literature, which points to the need for further quantitative research to assess associations of the identified factors with collaboration in IPCTs. More qualitative research is also needed to understand the complexity of the relationships among the various factors that influence human behavior in IPCTs. There may also be subtle differences that exist between the measures of collaboration used in the various studies. Finally, the proposed gears metaphor has some limitations. It is difficult to integrate the static concepts of the input-processes-output models with the dynamic nature of the interrelated factors in the proposed gears model and we encourage feedback and further development of this aspect of the gears metaphor. In addition, it is important not to suggest that providers are operating mechanically. This would fail to capture the creativity of teamwork. However, aligning contextual factors and processes so that these factors ‘click into gear’ can take the outcomes to levels that were not previously achieved.

The findings provide a solid foundation for future research to expand the empirical basis for the suggested factors. The conceptual framework points to the need for quantitative research to test the magnitude and statistical significance of economic, regulatory and political factors in the macro realm, organizational factors in the meso realm, and several formal and social process factors in the micro realm. It also suggests the need for additional qualitative research to explore the conceptual basis for these relationships in greater depth and in different contexts, as well as the mechanisms by which teams respond to changes in these factors. There is also a need for research that explores the role of patients as core members of the collaborative care team, going beyond how patient needs and preferences influence collaborative processes in an era of growing patient-centeredness. The identified studies did not address the role of patients in decision-making as part of the collaborative team as has been suggested elsewhere [19, 58]. We have attempted to capture this through the patient characteristics factor in the dynamic model of interprofessional collaboration, but greater clarity on how patient needs and preferences factor into the model and their measured associations should be a priority in future research. Finally, there is a need for additional research to better understand the relationship between interprofessional collaboration and end outcomes in terms of patient health, cost and quality outcomes.

## Conclusion

As jurisdictions around the world advance IPCT models of care delivery, the evidence suggests that key decision-makers can contribute in different ways to foster environments for improved interprofessional collaboration. The evidence is clear that within health care teams, formal and social processes and team structure are critical considerations. Many straightforward actions can be taken at this level, such as dedicating human resources to championing collaboration, setting a common vision and goals, attending to formal and social processes to minimize conflict and value the contributions of team members. Ongoing reflection for continuous improvement of the full team is required, through formal mechanisms like quality audits, as well as regularly scheduled team meetings.

At the same time, the context within which the team operates is important, although understudied. Based on this review, more research is needed to understand how policy and organizational contexts affect the ability of teams to collaborate effectively and how dynamic changes in these contexts influence collaboration within the team. A key lesson of the gears model is that collaboration is needed beyond the team itself, and must include actions of policy-makers, organizational leaders, health care team leads and individual professionals if IPCTs are to realize their promise of bettering address patient needs, particularly those with chronic and complex conditions.

## Abbreviations

IPCTs, interprofessional primary care teams; PRISMA, Preferred Reporting Items for Systematic Reviews and Meta-Analyses

## Additional file

Additional file 1: Sample Medline Search. Contains sample search terms and strings used for the Medline searches. Similar searches were performed for other databases. (DOCX 15 kb)

## Footnotes

1 Note that team size was not found to be associated with interprofessional collaboration in the study by Poulton and West (1999).

2 Having support from supervisors was not associated with team coordination in the study by Gené-Badia et al. (2007).

3 Note that the study Gené-Badia et al. (2007), being open to team members proposals was not associated with interprofessional collaboration.
