# Supplementary material for: ‘Gearing Up’ to improve interprofessional collaboration in primary care: a systematic review and conceptual framework
Source: BMC Fam Pract. 2016 Jul 20;17:83. doi: 10.1186/s12875-016-0492-1 (PMC4955241; doi:10.1186/s12875-016-0492-1)
Supplement: Additional file 1: — Sample Medline Search. Contains sample search terms and strings used for the Medline searches. Similar searches were performed for other databases. (DOCX 15 kb) [file 12875_2016_492_MOESM1_ESM.docx]

**Medline search terms**

**IPC general terms**

1. interprofessional cooperation.mp

2. inter-professional cooperation.mp

3. multidisciplinary cooperation.mp.

4. interdisciplinary cooperation.mp.

5. team based health.mp.

**Factor: Team Processes**

6. team process*.mp.

7. participatory management

8. participation.mp.

9. innovati*.mp.

10. decision making.mp. or decision making/

11. communication.mp. or interpersonal communication/

12. technology.mp or information technology/ or technology/

13. group process/ or team meeting*.mp

14. cooperation*.mp or cooperation/

15. "scope of practice".mp or "scope of practice"/

16. professional regulation*.mp

17. team culture*.mp

18. power*.mp

19. trust/ or trust*

20. practice guidelines/ or guidelines*.mp

21. communication protocol/ or protocol*.mp

22. accountabili*.mp

23. conflict management/ or conflict resolution*.mp

24. mutual respect*.mp

25. workload/ or balance workload.mp

26. time management/ or time/

27. training/ or training*.mp

28. professional supprt.mp

**Factor: Team Structure**

29. team structure*.mp

30. leadership/ or leadership*.mp

31. "cost control"/ or physical resource*.mp

32. financial management/

33. hierarchy.mp

34. goverance.mp

35. autonomy .mp.

36. shared goal*.mp.

37. fee/ or remuneration*.mp.

38. co-location*.mp.

39 colocation*.mp.

40 team size.mp.

41 professional isolation*.mp.

42 compensation/ or "salary and fringe benefit"/ or compensation model*.mp.

43 models of care.mp.

44 business plan*.mp.

45 cohesiveness.mp.

46 role clarity .mp.

**Outcome Factors:**

47. team effective*.mp

48. team satisfaction.mp

49. team performance*.mp

50. collaaboration*.mp

**Combination Searches**

51. 1 or 2 or 3 or 4 or 5 (IPC Factors)

52. 6 or 7 or 8 or 9 or 10 or 11 or 12 or 13 or 14 or 15 or 16 or 17 or 18 or 19 or 20 or 21 or 22 or 23 or 24 or 25 or 26 or 27 or 28 (Team Factors)

53. 29 or 30 or 31 or 32 or 33 or 34 or 35 or 36 or 37 or 38 or 39 or 40 or 41 or 42 or 43 or 44 or 45 or 46 (Team Structure Factors)

54. 47 or 48 or 49 or 50 (Outcome Factors)

**Combine Sets**

55. 51 and 52 and 53 and 54 5

56. Limit to yr="1990-2013" 4
